# Comparative Meta-Analysis of Transcriptomics Data during Cellular Senescence and *In Vivo* Tissue Ageing

**DOI:** 10.1155/2015/732914

**Published:** 2015-04-21

**Authors:** Konstantinos Voutetakis, Aristotelis Chatziioannou, Efstathios S. Gonos, Ioannis P. Trougakos

**Affiliations:** ^1^National Hellenic Research Foundation, Institute of Biology, Medicinal Chemistry and Biotechnology, 48 Vassileos Constantinou Avenue, 11635 Athens, Greece; ^2^Department of Cell Biology and Biophysics, Faculty of Biology, University of Athens, Panepistimiopolis, 15784 Athens, Greece

## Abstract

Several studies have employed DNA microarrays to identify gene expression signatures that mark human ageing; yet the features underlying this complicated phenomenon remain elusive. We thus conducted a bioinformatics meta-analysis on transcriptomics data from human cell- and biopsy-based microarrays experiments studying cellular senescence or *in vivo* tissue ageing, respectively. We report that coregulated genes in the postmitotic muscle and nervous tissues are classified into pathways involved in cancer, focal adhesion, actin cytoskeleton, MAPK signalling, and metabolism regulation. Genes that are differentially regulated during cellular senescence refer to pathways involved in neurodegeneration, focal adhesion, actin cytoskeleton, proteasome, cell cycle, DNA replication, and oxidative phosphorylation. Finally, we revealed genes and pathways (referring to cancer, Huntington's disease, MAPK signalling, focal adhesion, actin cytoskeleton, oxidative phosphorylation, and metabolic signalling) that are coregulated during cellular senescence and *in vivo* tissue ageing. The molecular commonalities between cellular senescence and tissue ageing are also highlighted by the fact that pathways that were overrepresented exclusively in the biopsy- or cell-based datasets are modules either of the same reference pathway (e.g., metabolism) or of closely interrelated pathways (e.g., thyroid cancer and melanoma). Our reported meta-analysis has revealed novel age-related genes, setting thus the basis for more detailed future functional studies.

## 1. Introduction

The lifetime of complex multicellular organisms includes embryogenesis (a highly programmed period) and the lifetime after birth, which is marked by the constant exposure to distinct types of stressors that gradually promote the stochastic damage of most cellular biomolecules [1, 2]. Due to the action of both quality control and clearance systems, organisms retain for a relatively long time low levels of damaged biomolecules but eventually, as the organism gets older, these homeostatic mechanisms are either compromised or disrupted, resulting in impaired signalling and repair or clearance pathways. These effects result in deteriorating cellular functions that correlate with increased disability, morbidity, tissue ageing, and inevitably death [3, 4]. In line with this view, age is the major risk factor for several diseases, including cardiovascular disease, cancer, neurodegeneration, and diabetes [5, 6].

Age-related accumulation of damaged biomolecules affects both the mitotic (e.g., epithelial, stromal, vascular, and haematopoietic stem cells) and the highly differentiated postmitotic cell lineages (e.g., neurons and skeletal muscle cells) [7]. Mitotic cells, which comprise the renewable tissues and organs of the human body, namely, the skin, intestines, liver, kidney, and so on [8], gradually lose their replicative potential and inevitably stop proliferating, as a result of serial passaging in tissue culture; this process is referred to as replicative senescence (RS) and in normal human cells relates to progressive telomere shortening, due to the absence of the telomerase (hTERT) gene expression [9, 10]. Young normal human cells having long telomeres may also senesce prematurely if exposed to various types of stress, during a process termed as Stress-Induced Premature Senescence (SIPS) [5]. It is assumed that a combination of both RS and SIPS contributes to human cells senescence* in vivo* [2], while a number of cellular senescence markers have been detected in various animal tissues and correlate with chronological ageing [11–13]. In addition, it has been shown that metabolites and secretory factors from senescent cells, such as proinflammatory cytokines, chemokines, growth factors, and proteases, contribute to various physiological malfunctions and may play a causative role in ageing or age-related diseases [2, 8].

Several signalling pathways have been functionally involved in the progression of cellular senescence and* in vivo* ageing including nutrients and energy sensing pathways, stress responsive pathways, as well as sirtuins, the rate of respiration, telomeres length, signals from the gonads, altered intercellular communication, exhaustion of stem cells, and epigenetic modifications [1, 2, 14–16]. Notably, most of these pathways have not been evolved as direct regulators of ageing as, for instance, nutrients signalling is critical in promoting growth effects during embryogenesis and early development [17].

Various studies have attempted, through high-throughput genome-wide transcriptomics, to identify gene expression signatures that define cellular senescence and/or* in vivo* tissue ageing. Nevertheless, comparative meta-analyses of senescence- and/or* in vivo* ageing-related transcriptomics data are scarce. Thus, in this study we performed a stringent bioinformatics meta-analysis of transcriptomics data from five cell- and seven biopsy-based microarrays experiments that include mitotic and postmitotic cell lineages and refer to both cellular senescence and* in vivo* ageing. Our goal was to reveal potential biomarkers of ageing, as well as common molecular pathways that characterize this complicated (and largely stochastic) biological process. We have succeeded to identify gene expression signatures and pathways alterations that mark cellular senescence, skeletal muscle, and neuronal ageing and also to reveal molecular commonalities between cellular senescence and tissue* in vivo *ageing.

## 2. Materials and Methods

### 2.1. Data Description

The selection of the transcriptomics data for our meta-analysis study was based on three criteria. The first one referred to the inclusion of data solely from human samples, while the second one referred to the quality of the deposited data in the database; in this case, only datasets that were fully annotated with a detailed description of the array platform were used. The last criterion was the origin of the transcriptomics data since we included in the study both mitotic and postmitotic cell lineages undergoing cellular senescence or* in vivo* ageing.

### 2.2. Cell-Based Gene Expression Transcriptomics Data

Cell-based transcriptomics data have been retrieved from five high-throughput microarrays experiments of different cell types that have been deposited in GEO as GSE24810 [18], GSE19018 (http://www.ncbi.nlm.nih.gov/geo/query/acc.cgi?acc=GSE19018), GSE15829 [19], GSE13496 [20], and GSE4352 [21]. Based on the cell types used, the five datasets (series) were grouped into four categories, namely, (a) Human Diploid Fibroblasts (HDFs) which include the HMF3A and HMF3S cells of GSE24810, the IMR90 cells of GSE19018, the HF cells of GSE15829, and the WS1, WI38, and BJ cell lines of the GSE4352 series; (b) Haematopoietic Progenitor/Stem Cells (HSCs) including the BMCD34^+^ and PBCD34^+^38^−^ cells of GSE13496; (c) T-cells of GSE13496; and (d) Human Mammary Epithelial Cells (HMECs) that include the 48R and 184 epithelial cells of the GSE4352 series.

### 2.3. *In Vivo* Gene Expression Transcriptomics Data

Transcriptomic data from* in vivo* studies have been retrieved from seven high-throughput microarrays experiments referring to nerve and muscle biopsies from young and aged healthy donors. These data have been deposited as six GEO DataSets, namely, GDS707 [22], GDS473, [23], GDS472 [23], GDS288 [24], GDS287 [24], and GDS156 [25], and in one GEO SEries set (GSE5086) [26]. All records (except GDS707 that grouped in the “Neuron” set) were grouped into the “Skeletal Muscle” set which (on the basis of the gender of the donors) was further subdivided into three groups, namely, “F” (females; GDS472, GDS473), “M” (males; GDS287, GDS288, and GDS156), and “F-M” (female and male donors; GDS156).

### 2.4. Computation Methods for the Estimation of Gene Expression among Different Experiments and/or Microarrays Platforms

For the transcriptomics data from cell-based experiments (except GSE15829 for which raw intensity values were not available in GEO), we implemented preprocessing methods in order to estimate and/or normalize gene expression levels. Background correction, spot quality filtering, log_2_ transformation, and normalization were the first preprocessing steps to homogenize retrieved data and compensate for systematic error measurements among different experimental platforms. These steps were performed by “Gene ARMADA” (Automated Robust Microarray Data Analysis), a MATLAB implemented, microarray, statistical analysis platform, equipped with a graphical user interface (GUI) (http://www.grissom.gr/armada/) [27]. For the GSE24810, GSE19018, and GSE13496 series (Affymetrix microarrays) the ∗.CEL files (Cell Intensity Files) were imported into the ARMADA platform, while samples of the GSE4352 dataset were imported as ∗.txt tab delimited files. The number of experimental conditions in ARMADA was defined as two, namely, “young” and “old”; in cases where data from quiescent cells were available, they were used to exclude genes that were coregulated in both cellular senescence and quiescence.

For Affymetrix GeneChip microarrays' background adjustment, normalization, and summarization, the GC-RMA algorithm was used (GC-Robust Multiarray Average) [28–30]; this involves optical correction and gene specific binding correction. The maximum likelihood method was used for signal estimation with tuning parameter set at 5 and number of steps (MLE) at 128. The Detection Call method of MAS5.0 algorithm [31, 32] was also implemented to characterize the Affymetrix probe sets as “Present (P),” “Marginal (M),” or “Absent (A)” using the default analytic parameters (Alpha 1: 0.04; Alpha 2: 0.06; and Tau: 0.015); marginal probe sets were considered as absent in order to reduce the rate of false positives. In the case of GSE4352 (a two-channel cDNA microarray platform), the Signal to Noise Ratio method for background correction was used [33]. Spot filtering was performed based on background noise to exclude spots whose mean signal intensity was less than 2-fold of the mean background intensity [34]. As a normalization method, the Robust Linear LOWESS model [35] with spanning neighbourhood at 0.1 was used [36, 37]. Student's *t*-test with a *P* value cut-off of 0.05 was employed for outlier detection among replicates of the same condition in both Affymetrix and cDNA arrays.

In biopsy-based transcriptomics data (GDS707, GDS473, GDS472, GDS288, GDS287, and GDS156 series), the detection *P* value threshold of 0.1 of Absolute or Detection Call of MAS4.0 (Affymetrix Technical Support, 2001) or MAS5.0 algorithm, respectively, was used in order to characterize each probe set as “Present (P)” or “Absent (A).” Transcripts with a detection *P* value ≥ 0.1 were considered as absent and their values were set to NaN (Not a Number), because Gene ARMADA handles these values as not detectable probes. In order to reduce the False Discovery Rate, if a probe set was labelled as “A” in more than 40% of samples of an experimental condition, it was marked as absent in all samples of this condition [25]; further, if a probe set was labelled as absent in all experimental conditions it was excluded from downstream analyses. For the dataset GSE15829 of the CodeLink platform, we used the assigned quality flags to remove probes that had been labelled as “MASK” and “Blank or Control” as well as those with a negative signal, keeping only spots with a good (G) quality flag. The probe list was also filtered by using the Negative Control Threshold of CodeLink data analysis guide from Applied Microarrays (http://www.appliedmicroarrays.com/index.php?option=com_content&view=article&id=11&Itemid=17). Finally, the processed data were imported into ARMADA as normalized expression values of 1 or 2 channels.

Differences between gene expression distributions among different slides of an experimental condition were evaluated by boxplots' comparison; quantile normalization was also performed if necessary. After “between slide” scaling, the k-nearest neighbour (kNN) algorithm was used to impute any missing value caused by the image processing or the filtering steps. The Euclidean distance was used as distance metric among gene vectors and the number of nearest neighbours was set to 10 [38]. Before the statistical test by ARMADA for the extraction of differentially expressed gene lists among different experimental conditions, the Trust Factor (TF) cut-off for each experimental condition (TF = #Appearances/#Replicates) was set to 0.6. In this case, a probe was marked as “Present” and its expression value was included in statistical analysis if it was “Present” at more than 60% of the samples of this condition; otherwise it was marked as “Absent.” If a probe was absent in all conditions, it was excluded from further analyses. For already processed biopsy-based transcriptomics, the TF was set to zero and thus all values were included in statistical analysis. Data were log_2_ transformed to comply with the normality assumption. Finally, Student's *t*-test with a *P* value ≤ 0.05 was adopted as the statistical selection, of the logarithmic signal values, in order to reveal genes, which are significantly differentiated between “young” and “old” experimental conditions (analyzed transcriptomics data and experimental conditions are summarized in Supplemental Table, S1, in Supplementary Material available online at http://dx.doi.org/10.1155/2015/732914). For multiple testing correction, the bootstrap approach of positive False Discovery Rate (pFDR; [39]) was used, with a mean FDR threshold of 0.1. Coupled to statistical testing, a fold change (FC) calculation was also applied in the list of significantly differentially expressed genes between “young” and “old” conditions, in order to filter out possible artefacts (false positives) of the preprocessing stage and highlight reliable biologically relevant changes; as a fold change of gene expression cut-off, the values of either 1.2- or (in most cases) 1.5-fold were used. The output of statistical processing including the FC in probe expression was exported to Excel spreadsheet files. Using the annotation file of each microarray platform, every probe ID was matched to a GenBank accession number and/or a HUGO gene symbol. When multiple transcripts matched the same gene ID, the average *P* value, average FDR, and average fold change were estimated; transcripts of the same gene with controversial pattern of expression were excluded from further analysis.

### 2.5. Mapping the Functional Implication of Recovered Genes in Signalling Pathways

Functional analysis was performed by statistical enrichment analysis using the StRAnGER (Statistical Ranking of Annotated Genomic Experimental Results) web-based application (http://mebioinfo.ekt.gr/stranger/home) [40]. StRAnGER exploits controlled biological vocabularies (e.g., the Gene Ontology or the KEGG pathways terms) in order to highlight significantly overrepresented biological processes (BP) and KEGG pathways [41] (http://www.genome.jp/kegg/pathway.html) involved in the molecular processes of ageing. For the series GSE15829 and GSE4352, the human genome annotation file provided by Ensembl (http://www.ensembl.org/info/genome/genebuild/genome_annotation.html) was used as background file since the annotation files of the platforms “CodeLink Human Whole Genome Bioarray” and “Cohen-31k1a” did not contain KEGG ontology terms. The ratio of the number of observations of a certain term in the list of significant findings* versus* the number of its observations in the whole array (or a more general reference list) is called the “enrichment score.” The functional enrichment results were corrected for multiple hypothesis testing following a bootstrap methodology as was previously described [40]. The bootstrap iterations were set in the default option (10000 iterations).

In order to further expand our knowledge regarding the potential, regulatory implications of “ageing-specific” genes in various cellular processes, we prioritized them according to their operational centrality (i.e., the estimation of their simultaneous functional attribution to different cellular processes) by exploiting Gene Ontology (GO) [42]. At the hierarchical structure of the respective graph tree, the candidate hub-genes were identified through GORevenge (Reverse engineering Gene Ontology networks) tool [43, 44] (http://mebioinfo.ekt.gr/goreveng/default/gr). Identification of significant genes was also performed in GORevenge through the use of the Resnik-BubbleGene algorithm with relaxation threshold set to 0.15 and pruning threshold set to 0.6 [44].

Data manipulation (i.e. sorting, filtering, comparisons, and heatmaps) of output files was performed in Excel 2003 and Excel 2007 and data retrieval was done by writing Perl scripts in ActivePerl 5.14.2 Build 1402 platform on Windows XP and Windows 7. Descriptive statistics were performed with GraphPad Prism version 5.00 for Windows (GraphPad Software, San Diego, CA USA). Venn diagram analyses for comparison between two and four datasets were implemented with the BioInfoRX Venn diagram plotter (http://bioinforx.com/free/bxarrays/venndiagram.php) and VENNTURE software [45], respectively.

## 3. Results

### 3.1. Age-Related Differential Gene Regulation in Nervous and Skeletal Muscle Tissues

Our initial studies in the nervous tissue (GDS707) dataset revealed a list of 395 significantly differentiated probe sets that correspond to 390 fully annotated genes with a HUGO gene symbol (Supplemental Table, S2). The biological processes affected were studied in a subset of 136 significantly differentiated genes (FC ≥ 1.5-fold) and were found to refer to synaptic transmission, signal transduction, protein transport, cellular component movement, calcium signalling pathway, regulation of actin cytoskeleton, phosphatidylinositol signalling system, and MAPK signalling pathway (Supplemental Table, S2).

**Figure 1:**
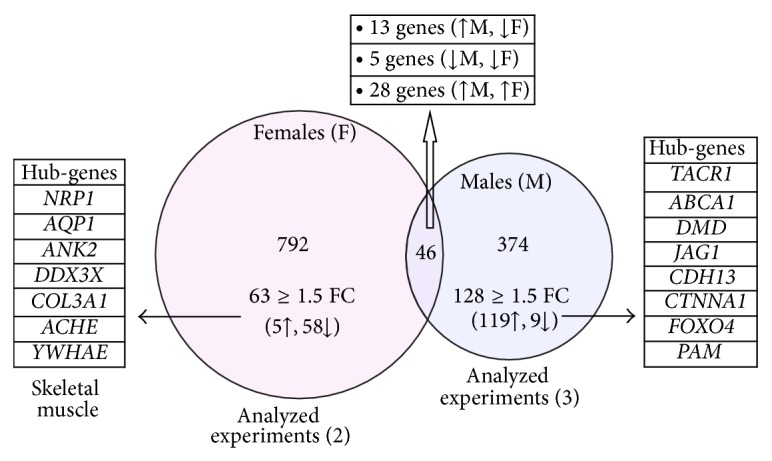
Identification of sex-specific age-related differentially expressed genes in human skeletal muscle. In skeletal muscle from aged males (M) (blue circle), 420 genes were found to be differentially expressed (*P* value ≤ 0.05; FDR ≤ 0.1; FC > 1.2), whereas 838 genes were differentially expressed in skeletal muscle from aged females (F) (pink circle). The intersection of the two groups refers to 46 genes; from these, 13 genes followed a sex-dependent expression pattern during ageing. The numbers of upregulated (↑) or downregulated (↓) DEGs along with the “hub-genes” linked to at least twenty Gene Ontology (GO) terms after pruning (revealed by the GORevenge algorithm) are indicated. The so-called “hub-genes” are linked to a plethora of Gene Ontology (GO) terms and are, likely, involved in numerous cellular procedures.

Parallel analyses in the skeletal muscle tissues datasets revealed sex-specific, age-related differences. Specifically, the majority of DEGs (119/128 ≈ 93%) with FC ≥ 1.5 in males appeared to be upregulated, whereas most of the differentially regulated DEGs in females (58/63 ≈ 92%) were downregulated. The 13 (out of the 46 common genes in males and females; Figure 1) that showed sex-dependent regulation are linked to GO terms involved in signal transduction, protein folding, apoptosis, cytokine-mediated signalling, RNA processing, calcium ion homeostasis, cell adhesion, and MAPK signalling. After enrichment analysis of (mostly downregulated) DEGs in female skeletal muscle samples, biological processes such as protein folding, apoptosis, transcription, ubiquitin-dependent protein catabolic process, and protein transport were found to be overrepresented. On the other hand, mRNA processing, RNA splicing, DNA replication, and DNA repair were overrepresented in males, where the majority of DEGs are upregulated (Figure 1; Supplemental Table, S3). The sex-specific genes that are differentially regulated during skeletal muscle ageing (FC ≥ 1.5-fold) are shown in Tables 1 and 2, respectively.

The common pathways (KEGGs) found to be affected significantly during skeletal muscle ageing in males and females related to oxidative phosphorylation, regulation of actin cytoskeleton, focal adhesion, MAPK signalling, and metabolism of carbohydrates. Oxidative phosphorylation was overrepresented in females (enrichment score: 25/104, *P* value 9.79*E* − 11), while in males it was the second most overrepresented pathway (enrichment score: 24/88, *P* value 1.04*E* − 11). Interestingly, the DEGs (and the corresponding pathways) identified to be implicated in the skeletal muscle ageing followed (except for carbohydrate metabolism) an opposite expression pattern in the two sexes as they were downregulated in females and upregulated in males. The prevalent pathways that were differentially affected during male skeletal muscle ageing related to Huntington's (HD), Alzheimer's (AD), and Parkinson's disease (PD) with the implicated genes being downregulated (Supplemental Table, S4). The insulin signalling pathway, which is central in ageing regulation, was deregulated only in the female skeletal muscle (enrichment score: 10/125, *P* value 0.0008) with eight genes being downregulated [*EIF4E*,* IKBKB*,* EIF4EBP1*,* SLC2A4*,* RHOQ*,* PHKA1*,* ARAF*, and* PRKAG1* (the full gene names are reported in Supplemental Table, S5)] and two genes (*INSR* and* PIK3R1*) being upregulated.

### 3.2. Comparative Analyses of Age-Related Gene Expression Signatures in the Postmitotic Nervous and Skeletal Muscle Tissues

**Figure 2:**
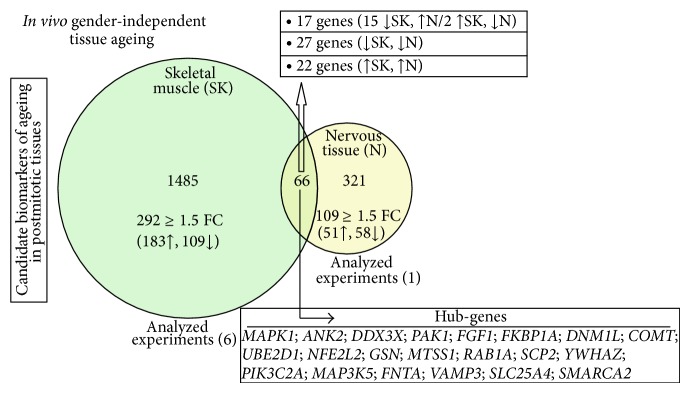
Identification of (gender-independent) coregulated potential biomarkers of ageing in human postmitotic skeletal muscle and nervous tissues. Venn diagram comparing DEGs in skeletal muscle and nervous tissues of young and aged individuals (*P* value ≤ 0.05; FDR ≤ 0.1; FC > 1.2). In skeletal muscle (light green; 6 experiments analysed), the total number of differentially expressed genes during ageing was 1551, while 387 genes were found to be differentially expressed during ageing of the nervous tissue (light yellow; 1 experiment analysed). The intersection of the two groups contains 66 common DEGs; the identified “hub-genes” (GORevenge algorithm) are listed in descending order of their GO terms' linkage number.

In order to identify genes that are regulated in an age-dependent manner in postmitotic tissues, we searched for common age-related alterations in both the skeletal muscle and nervous tissues (Figure 2). The* SEPP1*,* PIK3C2A*, and* NFE2L2* genes were found to be upregulated more than 1.5-fold in both tissues. On the other hand,* AZIN1*,* ANK2*,* DDX3X*, and* PAK1 *were downregulated in both tissues, while* SERINC5* was found to be upregulated in nervous and downregulated in the skeletal muscle.

In skeletal muscle, the KEGG pathways that were overrepresented (enrichment score: >20%, *P* value < 0.00001) were oxidative phosphorylation, citrate cycle (TCA cycle), and pyruvate metabolism, while the DEGs involved in these processes were downregulated. Genes encoding for the subunits of mitochondrial ATP synthase (e.g.,* ATP5B*,* ATP5G3*,* ATP5J*, and* ATP5C1*), cytochrome c oxidase (e.g.,* COX5A*,* COX5B*, and* COX6C*), succinate dehydrogenase (e.g.,* SDHB*,* SDHC*), NADH dehydrogenase (ubiquinone), (e.g.,* NDUFA8*,* NDUFA9*,* NDUFB5*, and* NDUFS1*), and the ubiquinol-cytochrome c oxidoreductase (e.g.,* UQCRB*,* UQCRC1*, and* UQCRH*) were deregulated in different datasets and highlighted KEGG pathways are involved in neurodegenerative diseases such as AD, HD, and PD. Following the descending order of enrichment score, three other affected KEGG pathways (in this case the vast majority of DEGs were upregulated) were proteasome (enrichment score: 19.4%, *P* value 0.0007), adherens junction (enrichment score: 17.3%, *P* value 2.31*E* − 05), and the Jak/STAT signalling pathway (enrichment score: 11.43%, *P* value 0.0008); the DEGs (>1.5-fold) implicated in these processes are listed in Supplemental Table, S4. At the nervous tissue, the phosphatidylinositol signalling system (enrichment score: 12.3%, *P* value: 1.24*E* − 07; DEGs were mainly downregulated), the calcium signalling pathway (enrichment score: 7.3%, *P* value: 4.98*E* − 07; DEGs mainly downregulated), and regulation of actin cytoskeleton (enrichment score: 6.5%, *P* value: 4.31*E* − 06; DEGs mainly upregulated) were the KEGG pathways with the higher enrichment scores. The DEGs (>1.5-fold) implicated in these biological processes are listed in Supplemental Table, S4.

**Figure 3:**
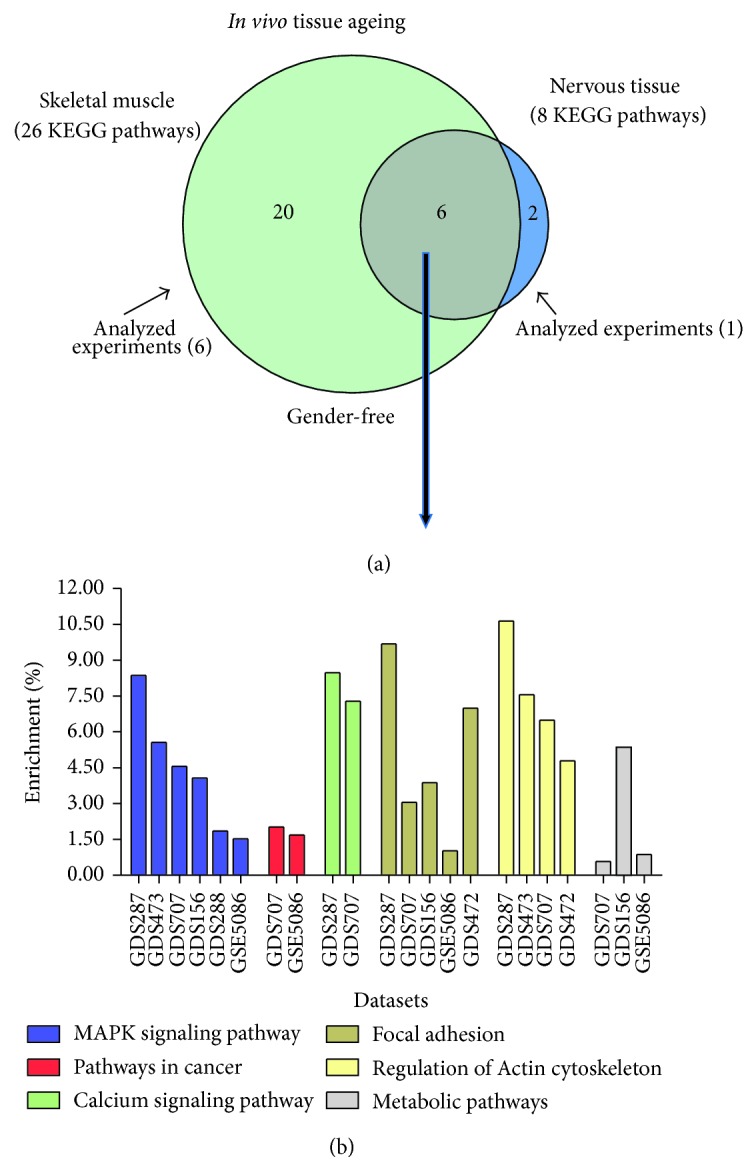
Identification of (gender-independent) age-related KEGG pathways that are coregulated in human postmitotic skeletal muscle and nervous tissues. (a) Venn diagram comparing differentially regulated KEGG pathways during ageing of human skeletal muscle and nervous tissues; data were assessed by the StRAnGER algorithm. The green circle represents the number of KEGG pathways as derived by the combinatorial analysis of six different experiments in skeletal muscle, whereas the blue circle represents the identified KEGG pathways as derived by the analysis of the GDS707 nervous tissue related dataset. The overlapping grey area represents those KEGG pathways that are commonly affected by ageing in the two tissues. (b) Distribution of the common overrepresented KEGG pathways in tissue datasets based on their (%) enrichment score. The (%) enrichment score represents the (%) ratio of the number of appearances of a KEGG ontology term in the list of DEGs versus the number which indicates the times that this KEGG ontology term exists in the annotation file of each microarray platform.

Finally, commonly regulated functions/pathways during ageing of the studied human postmitotic tissues (skeletal muscle and nervous tissues) included MAPK signalling, focal adhesion, regulation of actin cytoskeleton, metabolic pathways, calcium signalling, and pathways involved in cancer (Figure 3); the first three pathways were overrepresented in more than four of the datasets analysed (Figure 3(b); see also Supplemental Table, S4).

### 3.3. Genes and Molecular Signalling Pathways Affected by Cellular Senescence

The transcriptomics data obtained by models of cellular senescence were grouped into four different categories (see Section 2) based on the cell type. Analyses of the transcriptional alterations occurring during senescence of HFL-1 cells lead to the identification of 2532 DEGs (>1.2-fold); subsets of this list were represented by 1163 genes in HMF3A cells (GSE24810), 263 genes in IMR90 cells (cultured under 20% O_2_; IMR90 cells cultured in 3% O_2_ were not included in this analysis because under these conditions their replicative lifespan increases by ~30% [46–48]) (GSE19018), 237 genes in HF cells (GSE15829), 377 genes in WS1 cells (GSE4352), 25 genes in WI-38 cells (GSE4352), and 467 genes in BJ cells (GSE4352). The DEGs that were common between HMF3A and WS1 cells were 40, between HMF3A and BJ cells were 38 genes, and between HMF3A and IMR90 were 36 genes. The only common gene that was differentially expressed during senescence of BJs, HFL-1, IMR90, and HMF3A cells was* FBN2*. The biological processes that were overrepresented during cellular senescence of at least 50% of the different fibroblastic cell lines referred to signal transduction, cell adhesion, cell cycle regulation, protein amino acid phosphorylation, apoptosis, DNA repair, proteolysis, and RNA splicing. The molecular pathways associated with the aforementioned biological processes were focal adhesion, MAPK signalling, and pathways involved in cancer, while DNA replication and cell cycle scored the highest mean enrichment value (17.8% and 11.1%, resp.).

To determine whether ageing has similar impact on the gene expression of haematopoietic stem cells (HSCs), DEGs of human CD34^+^ cells from bone marrow (BMCD34^+^) and mobilized stem cell products (PBCD34^+^38^−^) were compared. Notably, no common genes were depicted between these cell lines and thus we included in our analyses DEGs of T-cells since they also derive from bone marrow pluripotent stem cells. Between PBCD34^+^38^−^ and T-cells, we found four common DEGs, namely,* C1QBP*,* TXN*,* PDIA6*, and* SERBP1*, whereas between T-cells and BMCD34^+^ we identified two commonly regulated DEGs, namely,* NUP50* and* PICALM*. Notably, among the KEGG pathways affected in T-cells and BMCD34^+^ cells were type I diabetes mellitus and the* Vibrio cholerae* infection pathway.

Concerning HMECs (cell lines 184 and 48R), only the* UPP1* gene was found to be in common among the identified cellular senescence-related DEGs. In these datasets, the majority (63.1%) of identified DEGs involved were downregulated and are involved in functions such as cell proliferation, mitosis, RNA metabolism, and apoptosis; on the other hand, the upregulated genes were mostly involved in blood clotting and platelet activation.

**Figure 4:**
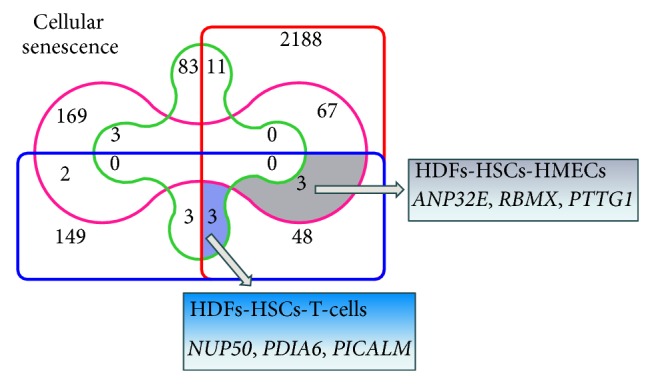
Venn diagram highlighting unique or shared DEGs during cellular senescence of the four human cell lines assayed. The numbers of differentially expressed genes (*P* value ≤ 0.05; FDR ≤ 0.1; F.C > 1.2) in HDFs (red frame); HSCs (blue frame); T-cells (green frame); and HMECs (purple frame) during cellular senescence are indicated. The two shaded intersections represent commonly regulated DEGs (respective genes are listed in each case) during cellular senescence of HDFs-HSCs-HMECs and of HDFs-HSCs-T-cells, respectively.

Finally, in order to further extend our statistical and functional meta-analysis, we tried to identify genes whose differential expression marks cellular senescence in a cell-independent manner (Figure 4). Our analyses revealed that HDFs and T-cells share 11 common DEGs, HDFs and HMECs 67 DEGs, T-cells and HMECs 3 DEGs, HDFs and HSCs 48 DEGs, and finally T-cells and HSCs 3 DEGs; the only DEGs that were common in more than two cell lines were the six genes shown in Figure 4. The functional analyses of cellular senescence-related DEGs in the four cell types revealed KEGG pathways, such as Huntington's and Parkinson's disease, cell cycle, focal adhesion, DNA replication, purine-pyrimidine metabolism, actin cytoskeleton, proteasome, and oxidative phosphorylation.

### 3.4. Comparison of Cell- (Cellular Senescence) and Biopsy-Based (*In Vivo* Tissue Ageing) Transcriptomics Data

**Figure 5:**
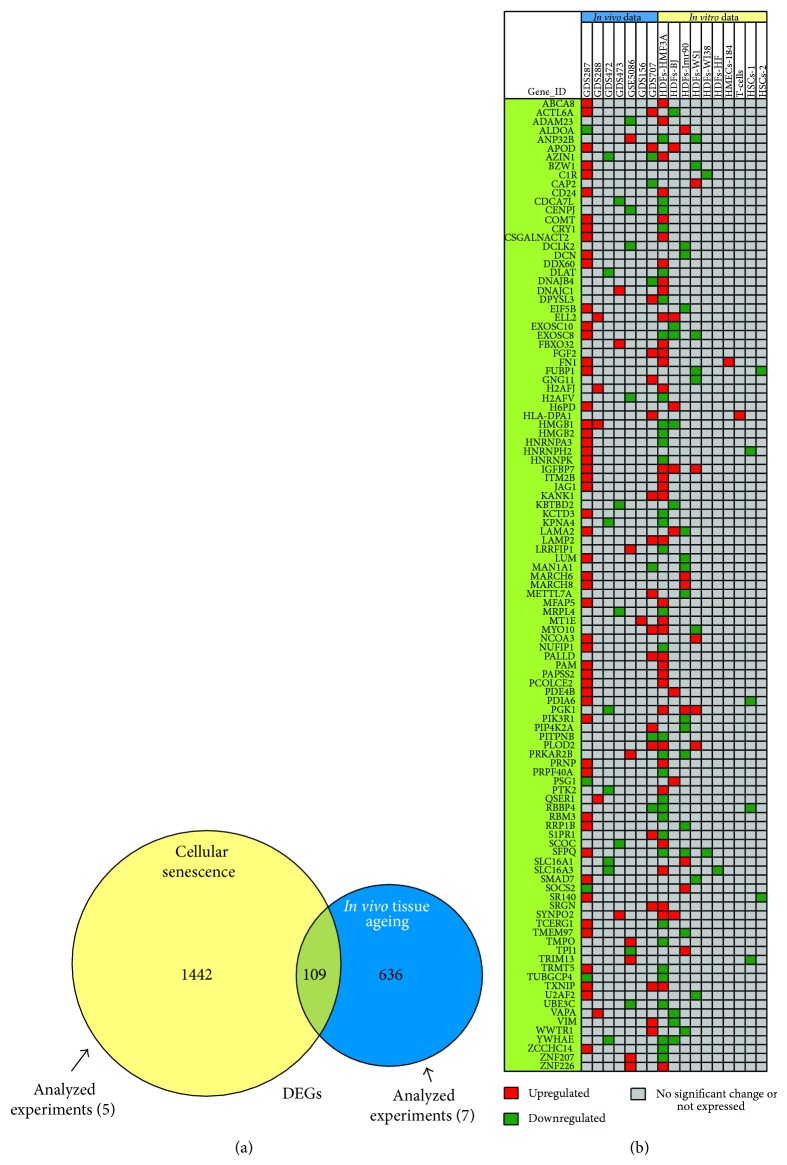
Differentially regulated human genes during cellular senescence and* in vivo* tissue ageing. (a) Venn diagram showing the differentially regulated genes (*P* value ≤ 0.05; FC ≥ 1.5) during cellular senescence and* in vivo* tissue ageing. The yellow circle represents the number of DEGs derived by the merging of five different datasets (experiments) of the cell-based transcriptomics data, while the blue circle represents the number of DEGs derived by the merging of seven different datasets (experiments) of* in vivo* tissue ageing transcriptomics. The overlapping “green” area indicates the number of DEGs that are common in both conditions. (b) Two-way hierarchical clustering analysis based on the expression profiles of the common differentially expressed genes. Each column represents the gene expression levels per dataset and each row denotes the corresponding gene. Red and dark-green colours indicate that the obtained values are greater than 1.5-fold or less than −1.5-fold, respectively; grey colour indicates statistically not significant changes or not expressed genes. Gene symbols are given in the left. HSCs-1: PBCD34^+^38^−^. HSCs-2: BMCD34^+^.

In order to identify human genes and/or molecular pathways, which are modulated by both cellular senescence and* in vivo* tissue ageing, we compared cell- and biopsy-based microarrays datasets. The numbers of DEGs during cellular senescence and* in vivo* tissue ageing, as well as the common genes between these two conditions, are summarized in Figure 5; the decreased number of DEGs shown in Figure 5 (as compared to Figures 2 and 4) is due to the more stringent fold change cut-off (≥1.5) that was applied. We found that 14.63% and 7.03% of DEGs that were identified during* in vivo* ageing and cellular senescence, respectively, were common. Notably, several DEGs (53.8%) displayed an opposite expression profile in the two biological settings. Furthermore, the genes identified to be involved in cellular senescence in a cell-type independent manner (Figure 4) were not found among the genes affected by* in vivo* tissue ageing (Figure 2). The majority (75.2%) of the 109 common genes were overexpressed in males' skeletal muscle and in human brain and were overrepresented in senescent HDFs.

The biological processes affected during both cellular senescence and* in vivo* tissue ageing were RNA processing (*P* value < 0.001; 13 genes), glucose catabolic process (*P* value < 0.001; 5 genes), cell migration (*P* value < 0.01; 6 genes), cell adhesion (*P* value < 0.05; 5 genes), response to hormone stimulus (*P* value < 0.01; 8 genes), DNA repair (*P* value < 0.05; 2 genes), negative regulation of cell development (*P* value < 0.05; 3 genes), and blood vessel morphogenesis (*P* value < 0.05; 5 genes). To further investigate whether the common DEGs are enriched for genes associated with certain KEGG pathways, the execution of StRAnGER algorithm revealed four KEGG pathways, namely, spliceosome (*P* value < 0.05; 6 genes), glycolysis/gluconeogenesis (*P* value < 0.05; 4 genes), small cell lung cancer (*P* value < 0.05; 4 genes), and the systemic lupus erythematosus (*P* value = 0.05; 4 genes).

**Figure 6:**
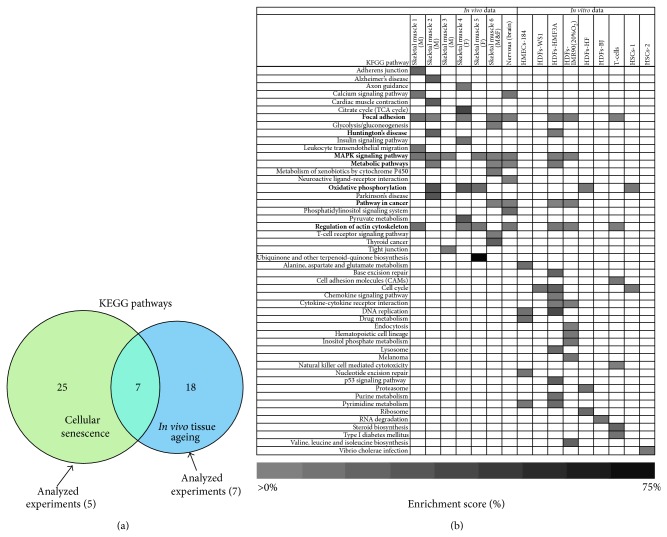
Significantly enriched KEGG pathways, which are deregulated during human ageing. (a) Venn diagram of KEGG pathways identified as overrepresented among the transcripts significantly up- or downregulated (*P* value ≤ 0.05; FDR ≤ 0.1) in cell- and biopsy-based transcriptomics; only KEGG pathways with count-threshold ≥2 (minimum number of genes for each KEGG pathway) were considered. The green circle represents the total number of KEGG pathways as derived by the combination of five different experiments, whereas the blue circle represents the number of KEGG pathways as derived by the analysis of seven different experiments; the overlapping area represents the common KEGG pathways (indicated in bold in Figure 6(b)). (b) Heatmap of the enrichment (%) score of predicted KEGG pathways in the context of the five cell- and seven biopsy-based datasets. Columns represent the enrichment score (%) of KEGG pathways per dataset and rows indicate the individual KEGG pathway; empty cells denote statistically not significant or no overrepresented KEGG pathway. M: male; F: female; skeletal muscle 1 (M): GDS287; skeletal muscle 2 (M): GDS156; skeletal muscle 3 (M): GDS288; skeletal muscle 4 (F): GDS472; skeletal muscle 5 (F): GDS473; skeletal muscle 6 (M, F): GSE5086; HSCs-1: PBCD34^+^38^−^ cells; HSCs-2: BMCD34^+^ cells.

By analyzing the overlap of cellular senescence and tissue ageing affected KEGG pathways, we found that this overlap comprises seven KEGG pathways that showed a high enrichment score (Figure 6); these were MAPK signalling (overrepresented in 6 out of 7 biopsy-based datasets and in 2 out of 5 cell-based studies), focal adhesion (overrepresented in 5 out of 7 biopsy-based datasets and in 3 out of 5 cell-based studies), regulation of actin cytoskeleton (overrepresented in 4 out of 7 biopsy-based datasets and in 3 out of 5 cell-based studies), metabolic pathways (overrepresented in 3 out of 7 biopsy-based datasets and in 2 out of 5 cell-based studies), oxidative phosphorylation (overrepresented in 3 out of 7 biopsy-based datasets and in 2 out of 5 cell-based studies), Huntington's disease (overrepresented in 1 out of 7 biopsy-based datasets and in 1 out of 5 cell-based studies), and cancer (overrepresented in 2 out of 7 biopsy-based datasets and in 2 out of 5 cell-based studies).

We also noted that most of the KEGG pathways being overrepresented exclusively at the cell- or the biopsy-based datasets were modules of the same common reference pathway, such as the citrate cycle, glycolysis/gluconeogenesis, and the pyruvate metabolic pathways found at the biopsy-based datasets or the purine, pyrimidine, inositol phosphate, alanine, aspartate, and glutamate metabolic pathways that were found at the cell-based transcriptomics. Furthermore, they represent KEGG pathways that are closely interrelated such as the adherens and tight junction pathways (biopsy-based datasets) or the cell adhesion molecules pathway (cell-based datasets), which are both interrelated to the common focal adhesion pathway. Similarly, the thyroid cancer and melanoma pathways are modules of the common reference pathway “pathways in cancer.” On the other hand, we also found tissue- or cell type-specific overrepresented KEGG pathways, such as the “axon guidance,” “neuroactive ligand-receptor interaction,” and the “cardiac muscle contraction” pathways, that reflect functions of the neuromuscular system or the “natural killer cell mediated cytotoxicity” and the “*Vibrio cholerae* infection” found in the T- and BMCD34^+^ cells. It is noteworthy that although the biological processes “cell cycle” and “DNA replication” were found to be overrepresented in the majority of cell- and biopsy-based data (most genes were found to be downregulated), their corresponding KEGG pathways were statistically significant only in the cell-based datasets. Moreover, the “Lysosome” and “Proteasome” pathways (which participate in protein turnover and maintenance), along with the “RNA degradation” (which tunes gene expression), the “Base excision repair” (whch controls DNA damage), and the “p53 signalling pathway” (which exerts a dominant role in cellular senescence) were found to be differentially regulated specifically in the cell-based datasets.

## 4. Discussion

It is still elusive whether the molecular alterations that mark cellular senescence correlate with similar gene expression changes during* in vivo* tissue ageing. To address this issue, we have investigated the cellular senescence- or* in vivo *age-related gene expression profile of mitotic (HDFs, HSCs, T-cells, and HMECs) cell lineages and postmitotic skeletal muscle and nervous tissues. By conducting a meta-analysis of transcriptomics data derived from 12 microarray experiments, we demonstrate herein significant similarities in the age-related gene expression, biological processes, and KEGG pathways changes during cellular senescence and* in vivo *tissue ageing. Our findings validate previously reported results on microarrays experiments of ageing-related studies [18–22, 24–26] and meta-analyses of age-related gene expression profiles [49, 50], and they indicate potential biomarkers of human ageing; these putative biomarkers include genes that were found to be differentially expressed in both the muscle and nervous system (Figures 2 and 5(a)).

The majority of underexpressed genes during ageing in the nervous tissue are associated directly or indirectly with synaptic transmission and plasticity [51, 52] that affects learning and memory functions [53]. Genes encoding for neurotransmitter receptors such as* HTR2A*,* GRIN2A*, subunits of the GABA_A_ receptor, and KCNAB1 showed significantly reduced expression in samples from aged donors. Suppressed genes in the brain included genes that mediate synaptic vesicle release and recycling (e.g.,* SCAMP5*,* SYN2*, and* CRH*), as well as members of the calcium signalling pathway (e.g.,* CALM3*,* CAMK2A*, and* CACNB2*), the calcium-binding protein* CALB1*, the Ca^2+^ transporting plasma membrane* ATP2B2*, and the calcium-activated transcription factor* MEF2C* with a central role in neuronal differentiation and survival [54, 55]. Furthermore, members of the protein kinase C family (e.g.,* PRKCB*) and genes involved in vesicle/protein trafficking showed reduced expression during ageing, including Rab GTPases,* TGOLN2*,* DYNC1I1*, and* CLTB*. It is worth noting that many genes of the cytoskeleton and cell motility pathways were age-repressed such as* ACTA1*,* KIF1B*,* MAP1B*,* MAP2*,* MAPT*, and the RAN protein, which regulates the formation and organization of microtubule network; all these proteins are necessary for the stabilization of microtubules promoting the axonal transport [56]. In addition, reduced expression levels in the elderly were found for the* PAK1* and* PAK3* genes, which play a crucial role in neuronal cell fate, polarization, and migration and are implicated in neurodegenerative diseases [57, 58]. In the group of genes identified to be consistently and robustly underexpressed during nervous tissue ageing, we also found genes being involved in DNA repair and proteolysis, such as the* TOP2B* enzyme [59] and* KLHDC3* which interacts with cullin-3 ubiquitin ligase [60, 61].

In parallel to the aforementioned findings that reveal reduced functionality of neuron cells in the aged brain, we observed overexpression of genes involved in stress responses, repair, and apoptosis. Among them is* HSPA2*, a chaperone that inhibits aggregation and mediates folding of newly translated polypeptides [62], and* SEPP1*, that plays central role in selenium homeostasis [63, 64] and has been linked to glucose metabolism and type 2 diabetes [65], as well as several members of the ROS-activated MAPK family (e.g.,* MAPK1*,* MAP2K1*,* MAP3K4*, and* MAP4K3*) [66, 67]. Additional induced stress-related genes included* DDIT4* that regulates p53/TP53-mediated apoptosis in response to DNA damage via its inhibition on mTORC1 activity [68, 69];* GJA1*, which mediates the transduction of cell survival signals [70, 71]; and* LITAF*, which has been functionally involved in TNF-*α* and p53-induced apoptosis [72, 73] as well as in inflammatory and immune responses [74, 75]. These observations are in line with a previous meta-analysis study of age-related gene expression profiles by de Magalhães and coworkers [49] who found that the immune/inflammatory response pathway is upregulated during aging, highlighting also genes involved in stress responses and apoptosis. Thus, as these authors suggested [49], the identified gene expression signatures (though, likely, revealing true biomarkers of human ageing) may in fact indicate an indirect transcriptional response to the progression of ageing rather than an underlying mechanism; nevertheless, this notion should await further experimental evidence.

Regarding the sex-dependent gene expression signature in the aged skeletal muscle, most of the 46 common (though being differently regulated) DEGs in males and females are involved in fundamental biological processes and pathways, including oxidative phosphorylation, MAPK signalling, protein folding, apoptosis, cytokine-mediated signalling, and calcium ion homeostasis. These observations further support the notion that gender is a risk factor for a plethora of pathological conditions in humans including ageing [76–78]. These sex-dependent differences in expression profiles could be explained by documented variations in gene regulation [79, 80] or by the different physiological or biochemical requirements of the respective tissues in the two genders. Specifically, the reported sex-dependent differences in skeletal muscle overall mass and fatigability [81], the type of individual fibbers [82–85], the activities of several metabolic enzymes, the sex-dependent lipid content and oxidation [82, 86, 87], the relative expression of different myosin isoforms [88], and the distinct hormonal levels may impact age-related changes of the muscle [89] including muscle protein synthesis and differentiation [90]. On the basis of our findings, gene expression profile in males is characterized by overexpression of genes involved in transcription, mRNA processing, and translation process (these genes were suppressed in females) [80], something which might represent a reparative mechanism in the muscle to counteract the age-related declined protein synthesis and increased muscle break-down. Sex-dependent differential gene expression levels were also associated with the key longevity regulating pathway of insulin signalling in the skeletal muscle of aged females, where we noted suppression of the EIF4E,* IKBKB*,* EIF4EBP1*,* SLC2A4*,* RHOQ*,* PHKA1*,* ARAF*, and* PRKAG1* genes and overexpression of* INSR* and* PIK3R1*. Various studies have indicated that females are more sensitive to insulin as regards both the stimulation of glucose uptake in muscle and the suppression of glucose production in liver [91]; the defects in this pathway lead to various metabolic abnormalities such as type 2 diabetes, hyperlipidaemia, and cardiovascular diseases. On the other hand, genetic polymorphisms or mutations affecting (among others) the insulin receptor or downstream intracellular effectors such as AKT, mTOR, and FOXO have been linked to longevity in humans and model organisms [2, 92]. The prevalent pathways that were differentially affected during male ageing relate to neurodegenerative diseases such as HD, AD, and PD; in this case, the implicated genes (*CASP7*,* CALM3*,* CYCS*,* SDHC*, multiple subunits of Complex I, Complex IV, and ATP synthase) are downregulated. Our observations are supportive of previous studies implicating dysfunction of mitochondria [49, 93] and accumulation of reactive oxygen species (ROS) in the pathogenesis of progressive neurodegenerative diseases [94, 95].

Despite the limited number of common DEGs among the skeletal muscle and nervous tissues, our analyses have revealed commonly affected KEGG pathways; these include the MAPK signalling pathway, focal adhesion, regulation of actin cytoskeleton, metabolic pathways, the calcium signalling pathway, and pathways involved in cancer. These findings support the existence of common age-related gene expression signatures in the different tissues [49]; nevertheless, the low number of common DEGs indicate age-related gene expression patterns which are, likely, tissue-specific [26, 96]. On the basis of the existing commonalities, we can assume that different genes or/and members of protein families may operate as sensors of the ageing process by regulating common nodal hub-genes that have been evolutionary “chosen” to operate as conductors. These hub-genes could represent candidate targets for rejuvenating and pharmaceutical interventions aiming to improve human health during ageing.

In the case of cellular senescence, gene expression profiling of distinct cell types including epithelial, fibroblastic, vascular, and haematopoietic stem cells highlighted the great genetic heterogeneity that characterizes cellular senescence in culture, since the only gene that was differentially expressed during senescence of human fibroblasts was* FBN2*. FBN2 is secreted into the extracellular matrix (ECM), becomes incorporated into the insoluble microfibrils, and is likely involved in elastic fibber assembly [97]. Moreover, FBN2 has been associated with reduced TGF-*β*, expression, angiogenesis, and increased oxidative stress in dermal fibroblasts [98]. Concerning HMECs, only the* UPP1* gene was common among the 244 DEGs found in senescent cells;* UPP1* has been found overexpressed in various forms of solid cancers such as breast and ovarian cancer [99, 100]. DEGs that were common in more than two cell lines among the four cell types studied (Figure 4) have been associated with senescence phenotype and age-related diseases such as PD or AD. In particular,* NUP50* is involved in age-related deterioration of nuclear pore complexes, leading to increased nuclear permeability and influx of cytoplasmic proteins into the nucleus. This nuclear “leakiness” is dramatically accelerated during ageing, leading to the disruption of genome integrity. It has thus been associated with the increased oxidative damaged nucleoporins in old cells [101]. The protein PDIA6, an enzyme located in the endoplasmic reticulum (ER), catalyzing disulfide-bond formation, has a pivotal role in protein folding. The upregulation of its expression under ER stress in transgenic mouse models driven by the accumulation of unfolded proteins indicates a cytoprotective role for PDIA6 [102, 103]. Specific alleles of* PICALM* gene, encoding phosphatidylinositol-binding clathrin assembly protein, were recently shown to be associated with high risk of AD in the elderly [104–106]. The ANP32E, a protein phosphatase 2 inhibitor (particularly in brain tissue, together with Cpd1 regulating protein phosphatase 2A activity), has been found to be localised at synapses during synaptogenesis [107] acting in a complex with ANP32A and SET for the stabilization of short-lived mRNAs, containing AU-rich elements. Moreover, the ANP32E protein possesses an acetyl-transferase inhibitory activity (in a complex with SET protein), playing an important a role in chromatin remodelling and transcriptional regulation [108]. Finally, the* PTTG1* gene has been involved in cell cycle regulation through the inhibition of sister chromatid separation. Upregulation of* PTTG1* has been correlated with tumor formation and its induction in senescent fibroblasts correlates with activation of p53 pathway in response to DNA damage [109]. Furthermore, the identification of the “Lysosome” and “Proteasome” pathways (among those that are deregulated during cellular senescence of different cell types) is of interest as these machineries play a central role in protein homeostasis and their functionality declines with age [110, 111], while perturbations of the ubiquitin-proteasome pathway have been involved in the pathogenesis of various neurodegenerative diseases including AD and HD [112–115].

Interestingly, over 50% of the common DEGs between* in vivo* tissue ageing and cellular senescence exhibit a controversial expression profile with the majority of them being upregulated in postmitotic tissues and downregulated in senescent cells. Genes involved in transcriptional activation, chromatin remodelling, cell-matrix adhesions, signal transduction, inflammation, and repair processes had increased expression levels in tissue samples, highlighting the differential complexity that characterizes* in vivo* tissue ageing and cellular senescence. This is anticipated since the cells within a tissue are confronted with a plethora of stimuli and stress factors that derive from the extracellular environment and dictate genetic and epigenetic changes that could account for the observed differences. However, despite these differences, seven KEGG pathways were overrepresented in both conditions (i.e., cellular senescence and* in vivo* tissue ageing), namely, Huntington's disease, cancer, MAPK signalling, focal adhesion, regulation of actin cytoskeleton, metabolic pathways, and oxidative phosphorylation; these findings indicate that (at least to some extent) there are significant commonalities between cellular senescence and* in vivo* ageing with enriched pathways being involved in cell-extracellular matrix interactions and stress responses [116]. For instance, intercellular communication, as well as the interplay of cells with the extracellular matrix, is pivotal for the appearance of the senescent phenotype. This is supported by the fact that senescent cells can also induce senescence in neighbouring cells via gap junction-mediated cell-cell contacts and processes involving ROS [117]. Indeed, the cell-matrix adhesions were found to play essential role in our analyses. Integrin signalling is dependent upon the nonreceptor tyrosine kinase activities of the FAK (Focal Adhesion Kinases) and Src (protooncogene tyrosine-protein kinases) proteins, as well as the adaptor protein functions of FAK, Src, and Shc (Src homology 2 domain containing) to initiate downstream signalling events. These signalling events culminate in reorganization of the actin cytoskeleton: a prerequisite for changes in cell shape, motility, and gene expression [118]. However, FAK is activated not only by integrin engagement but also through stimulation by hormones and growth factors, including insulin and insulin-like growth factor-I (IGF-I) [119, 120]; the latter is being underexpressed during both cellular senescence and* in vivo* tissue ageing.

## 5. Conclusions

In conclusion, our presented studies have revealed several signalling pathways which are, likely, implicated in the molecular phenotypes of cellular senescence and/or* in vivo* tissue ageing; the clarification, however, of whether these alterations reflect responses to the progression of ageing or causal mechanisms (i.e., gerontogenes) should await further experimentation. Despite sex-dependent variability (which has been also reported previously in mouse studies analyzing caloric-restriction mediated effects [121]), a number of responses to signalling pathways which are functionally involved in cancer, focal adhesion, actin cytoskeleton, MAPK, and calcium signalling, as well as in metabolism regulation, were found to be differentially regulated during ageing of the skeletal and nervous postmitotic tissues. On the other hand, genes that are regulated in a cell type-independent manner during cellular senescence refer to pathways involved in neurological diseases, focal adhesion, actin cytoskeleton, proteasome, cell cycle, DNA replication, purine-pyrimidine metabolism, and oxidative phosphorylation. Coregulated pathways during cellular senescence and* in vivo* tissue ageing referred to cancer, Huntington's disease, MAPK signalling, focal adhesion, actin cytoskeleton, oxidative phosphorylation, and metabolic signalling. In summary, our reported meta-analysis has revealed novel sex- and tissue-biomarkers and biological processes that are functionally involved in the human ageing phenotype, setting thus the basis for more detailed future functional and validation studies.

## Supplementary Material

Supplementary material includes five (5) excel files termed as Supplementary Tables S1-S5.
The S1 file includes one (1) spreadsheet; a summary Table of analyzed transcriptomics data and experimental conditions.
The S2 file includes nine (9) spreadsheets: (1) a list of 390 fully annotated DEGs extracted from Statistical analysis (p-value≤0.05, FDR≤0.05, F.C filtering >0.26 and <-0.26, in log2 scale) of nervous tissue; (2) a list of DEGs in nervous tissue with gene expression-fold change >=1.5 fold; (3) a summary Table with the results of functional analysis of DEGs in nervous tissue by "Stranger" based on GO terms; the (4), (5), (6) Tables with the results of functional analysis of DEGs in nervous tissue with "Stranger" based on Cellular Location, Function and Biological Process of GO tree, respectively; (7) a list of DEGs enriched in Functions and Biological Processes found to be overrepresented in nervous tissue transcriptomics data; (8) a Table with the genes and GO terms associated with DEGs enriched in Biological Processes found to be over-represented in nervous tissue transcriptomics data - "Gorevenge output"; (9) a list of DEGs in nervous tissue transcriptomic data characterized as hub-genes from "Gorevenge" (in a descending order of linked GO terms).
The S3 file includes seven (7) spreadsheets: the (1), (2) Tables with the enrichment score of Functions and Biological Processes over-represented in males muscle tissue, respectively (the number of enriched up- or down-regulated genes is also shown); the (3), (4) Tables with the enrichment score of Functions and Biological Processes over-represented in females muscle tissue, respectively (the number of enriched up- or down-regulated genes is also shown); (5) a list of genes enriched in over-represented Biological Processes of females muscle tissue with the common genes and Biological Processes to be highlighted; (6) a Table with the enriched up- or down- regulated genes in over-represented Biological Processes of males muscle tissue (a comparative histogram of these numbers in three datasets is also depicted); (7) a Table with the enriched up- or down- regulated genes in over-represented Biological Processes of females muscle tissue (a comparative histogram of these numbers in three datasets is depicted).
The S4 file includes three (3) spreadsheets: (1) a cumulative list of DEGs during in vivo tissue ageing. DEGs specific in males (M) and females (F) are highlighted with blue and red color, respectively; DEGs common in both sexes (F, M) are shown with black lettering; (2) a summary Table with the overrepresented KEGG pathways and their enriched DEGs in nervous and skeletal muscle tissues during in vivo ageing; (3) a summary Table with the overrepresented KEGG pathways in nervous and skeletal muscle tissues during in vivo ageing.
The S5 file includes one (1) spreadsheet with a list of genes being cited in the manuscript (documents their full name).

## Figures and Tables

**Table 1:** Age-regulated genes in human males' skeletal muscle.

Function	Gene name	Gene description	Fold change	FDR
Glucose/lipid metabolism	H6PD	Hexose-6-phosphate dehydrogenase (glucose 1-dehydrogenase)	2.35	0.013
	ALDOA	Aldolase A, fructose-bisphosphate	0.12	0.062
	ATP5S	ATP synthase, H^+^ transporting, mitochondrial Fo complex, subunit s (factor B)	0.66	0.102
	ZDHHC11	Zinc finger, DHHC-type containing 11	2.04	0.032

Signal transduction/G protein signalling	LEPR	Leptin receptor	2.31	0.021
	LGR5	Leucine-rich repeat-containing G protein-coupled receptor 5	2.34	0.126

Stress response/redox homeostasis	ADH1B	Alcohol dehydrogenase 1B (class I), beta polypeptide	2.17	0.044
	BPHL	Biphenyl hydrolase-like (serine hydrolase)	0.59	0.027
	GSTT1	Glutathione S-transferase theta 1	0.63	0.108
	GSR	glutathione reductase	0.64	0.082
	DHRS4L2	Dehydrogenase/reductase (SDR family) member 4 like 2/dehydrogenase/reductase (SDR family) member 4	0.64	0.028
	HSPA2	Heat shock 70 kDa protein 2	1.91	0.092

Cell adhesion/extracellular matrix organization	LUM	Lumican	2.03	0.097
	MFAP5	Microfibrillar associated protein 5	2.17	0.053
	FN1	fibronectin 1	1.91	0.085

Unknown function	KIAA0240	KIAA0240	2.10	0.015
	ABHD3	Abhydrolase domain containing 3	2.34	0.034
	KIAA1107	Uncharacterized protein KIAA1107	0.56	0.069

Synaptic function	LIN7C	lin-7 homolog C (*C. elegans*)	1.94	0.015
	LRP1B	Low density lipoprotein receptor-related protein 1B	2.08	0.057
	FCER1A	Fc fragment of IgE, high affinity I, receptor for; alpha polypeptide	0.51	0.022
	MTMR3	Myotubular related protein 3	2.05	0.024
	DDN	Dendrin	2.05	0.04

Cell growth/myelination	ID4	Inhibitor of DNA binding 4, dominant negative helix-loop-helix protein	2.04	0.051
	YPEL1	Yippee-like 1 (*Drosophila*)	2.18	0.078
	CRIM1	Cysteine-rich transmembrane BMP regulator 1 (chordin-like)	1.96	0.094
	EMP1	Epithelial membrane protein 1	2.02	0.039

Inflammatory response	SOCS2	Suppressor of cytokine signalling 2	0.49	0.041
	TACR1	tachykinin receptor 1	0.61	0.024

Mitochondrial/translation	MRPS16	Mitochondrial ribosomal protein S16	0.51	0.036
	MRPL19	Mitochondrial ribosomal protein L19	1.92	0.015

Transcription	FRY	Furry homolog (*Drosophila*)	0.66	0.053
	ZFP36L2	Zinc finger protein 36, C3H type-like 2	2.02	0.015
	TET2	tet oncogene family member 2	1.9	0.091

Microtubule cytoskeleton	TUBB2A	Tubulin, beta 2A	1.9	0.043
	TNNC1	Troponin C type 1 (slow)	0.15	0.082
	TUBGCP4	Tubulin, gamma complex associated protein 4	0.61	0.025
	MAPT	Microtubule-associated protein tau	0.57	0.061

Muscle contraction	MYL1	Myosin, light chain 1	0.64	0.094

Apoptosis	DAPK3	Death-associated protein kinase 3	0.64	0.083

Shown genes are representative per functional group. Gene names by HUGO; fold changes [indicating differences in old versus young tissues (values <1 denote downregulation of the respective gene)] and statistical FDR values are indicated.

**Table 2:** Age-regulated genes in human females' skeletal muscle.

Function	Gene name	Gene description	Fold change	FDR
Lipid metabolism	GDE1	Glycerophosphodiester phosphodiesterase 1	0.55	0.035
	FABP3	Fatty acid binding protein 3, muscle and heart (mammary-derived growth inhibitor)	0.55	0.019
	ECHDC1	Enoyl CoA hydratase domain containing 1	0.57	0.034
	REPIN1	Replication initiator 1	0.59	0.078

Signal transduction/G protein signalling	NDRG2	NDRG family member 2	0.42	0.041
	PDE11A	Phosphodiesterase 11A	1.78	0.085
	TNK2	Tyrosine kinase, nonreceptor, 2	1.52	0.016
	SMAD9	SMAD family member 9	1.72	0.070
	CNKSR2	Connector enhancer of kinase suppressor of Ras 2	0.50	0.021
	SH3BP5	SH3-domain binding protein 5 (BTK-associated)	2.12	0.022

Stress response	HSPB6	Heat shock protein, alpha-crystalline-related, B6	0.58	0.029

Cytoskeleton	SYNPO2	Synaptopodin 2	1.88	0.045
	MYOM3	Myomesin family, member 3	0.51	0.018

Proteolysis	FBXO32	F-box protein 32	1.63	0.064
	SPG7	Spastic paraplegia 7 (pure and complicated autosomal recessive)	0.52	0.031
	DNAJC1	DnaJ (Hsp40) homolog, subfamily C, member 1	1.51	0.050
	TFRC	Transferrin receptor (p90, CD71)	0.50	0.026

Cell death/apoptosis	CABC1	Chaperone, ABC1 activity of bc1 complex homolog (S. pombe)	0.36	0.053
	YWHAE	Tyrosine 3-monooxygenase/tryptophan 5-monooxygenase activation protein, epsilon polypeptide	0.58	0.038
	SCOC	Short coiled-coil protein	0.54	0.037

Transmembrane transport	SLC16A3	Solute carrier family 16, member 3 (monocarboxylic acid transporter 4)	0.50	0.011
	TMED2	Transmembrane emp24 domain trafficking protein 2	0.56	0.024

Transcription	DNAJB6	DnaJ (Hsp40) homolog, subfamily B, member 6	0.53	0.038

	PHTF2	Putative homeodomain transcription factor 2	0.54	0.045

Mitochondrial/translation	MRPL4	Mitochondrial ribosomal protein L4	0.56	0.038

Shown genes are representative per functional group. Gene names by HUGO; fold changes [indicating differences in old versus young tissues (values <1 denote downregulation of the respective gene)] and statistical FDR values are indicated.

## Abbreviations

GEO: Gene Expression Omnibus
GO: Gene Ontology
HDFs: Human Diploid Fibroblasts
HSCs: Haematopoietic Progenitor/Stem Cells
HMECs: Human Mammary Epithelial Cells
F: Female
M: Male
DEGs: Differentially expressed genes
FDR: False Discovery Rate
FC: Fold change
AD: Alzheimer's disease
PD: Parkinson's disease
HD: Huntington's disease
BP: Biological process
ECM: Extracellular matrix.
